# Unemployment and hair cortisol as a biomarker of chronic stress

**DOI:** 10.1038/s41598-022-25775-1

**Published:** 2022-12-14

**Authors:** Mario Lawes, Clemens Hetschko, Ronnie Schöb, Gesine Stephan, Michael Eid

**Affiliations:** 1 Department of Education and Psychology, Freie Universität Berlin, Habelschwerdter Allee 45, 14195 Berlin, Germany; 2 Economics Department, Leeds University Business School, University of Leeds, Leeds, UK; 3 Department of Economics, School of Business & Economics, Freie Universität Berlin, Berlin, Germany; 4 CESifo, Munich, Germany; 5 Institute for Employment Research (IAB), Nuremberg, Germany; 6 School of Business, Economics and Society, Friedrich-Alexander-Universität Erlangen-Nürnberg, Nuremberg, Germany

**Keywords:** Psychology, Biomarkers, Risk factors

## Abstract

Unemployment is widely considered an important chronic stressor. Using longitudinal data of initially employed German jobseekers, the present study examines whether unemployment is related to changes in hair cortisol concentration (HCC), a reliable biomarker for chronic stress. The results indicate that HCC is the highest initially when individuals are insecurely employed and decreases as people gain certainty about whether they enter unemployment or not. We find no effects when comparing the average changes in HCC between individuals who entered unemployment to those of continuously employed individuals. However, medium-term unemployment was associated with a stronger mean increase in HCC if re-employment expectations were low compared to when re-employment expectations were high. Taken together, our results support two key conclusions. First, experiencing the uncertainty of looming unemployment is associated with more pronounced cortisol secretion than unemployment itself. Second, whether working or being unemployed is associated with higher HCC is highly context-dependent, with poor re-employment prospects during unemployment being a key predictor of increased HCC. Overall, our study provides further evidence that the physiological stress system is especially sensitive to uncontrollable situations and unfamiliar challenge.

## Introduction

*Stress* is the physiological and psychological response caused by internal or external stressors^1,2^. When an individual is confronted with a stressor, the organism initiates a physiological stress response in order to maintain homeostasis^3^. A central role in this physiological stress response is played by the hypothalamus–pituitary–adrenal (HPA) axis, which connects the central nervous system with the hormonal system. The main effector of the HPA axis is cortisol, a hormone that regulates critical physiological systems (e.g., the immune system) as well as affective and cognitive processes and mobilized bodily resources to overcome the increased demands resulting from the stressor^4,5^. While the acute physiological stress reaction is an adaptive mechanism of the organism, a dysregulated or prolonged cortisol secretion (e.g., following chronic stress) is related to numerous negative health outcomes^5,6^. For example, chronic stress (i.e., the response induced by long-term stressors) is linked to an increased risk for cardiovascular disease^7^, obesity^8^, type 2 diabetes^9^, reduced fertility^10^, as well as mental disorders^11–13^.

In our modern society, one’s economic situation is an especially potent stressor^14,15^. For example, a yearly survey in the US indicates that work, money and the economy are consistently named as the most common sources of stress^16^. Crucially, unemployment has further been linked to highly similar negative health outcomes as chronic stress^17–19^, suggesting that chronic stress and the subsequent over-production of cortisol might channel the long-lasting negative impact of unemployment on health. The present study partially probes this relationship by examining the effects of short- and medium-term unemployment on hair cortisol concentration (HCC), a reliable biomarker for chronic stress.

Past research indicated that unemployment has detrimental effects on physical, subjective, and mental well-being^20,21^. Specifically, it generally causes financial strain and is associated with a wide range of psychosocial stressors, such as a loss of identity^22,23^, feeling rejected during job search^24^ and growing uncertainty^25^. While most existing research on the effects of unemployment focused on subjective measures of well-being and mental health (e.g.^26–28^), only few studies examined the effects of unemployment on (physiological) stress. Existing studies using self-reported stress data found that unemployed individuals generally report higher levels of perceived stress than employed individuals^25,29,30^. Importantly, however, empirical studies on the relationship between unemployment and cortisol as a biomarker for stress are scarce, mostly cross-sectional and provide mixed results^15^. Further, existing studies generally rely on acute measures of cortisol secretion based on plasma or saliva samples which are less useful for studying chronic stress exposure^5,31^. Across studies utilizing measures of acute cortisol secretion (i.e., blood or saliva samples), some reported higher overall levels of cortisol in unemployed individuals compared to employed individuals^32^, others found no differences between these groups^33,34^ and one found higher overall cortisol levels among employed individuals in comparison to unemployed individuals^35^.

In recent years, the analysis of hair cortisol concentration has become the gold standard method of obtaining a reliable and valid biomarker of chronic stress^31^. As human hair grows at a fairly predictable rate of 1 cm per month^36^, aggregated cortisol levels over multiple months can be examined retrospectively. The accumulated cortisol secretion in hair over 1 month was shown to be highly correlated with the 30-day average across three daily saliva samples within the same period^37^. There is only one study in the context of unemployment that used HCC as a biomarker for chronic stress. This study found that average HCC levels of long-term unemployed individuals (> 12 months) were significantly higher than those of employed individuals^38^. Yet, due to the cross-sectional nature of this study and the rather selective sample, longitudinal studies analyzing HCC levels of individuals before and after entering unemployment are needed.

Our study fills this gap and extends the existing literature in three important ways. First, we utilize prospective panel data on HCC of initially employed German jobseekers who were at high risk of losing their job. Unlike existing studies that relied on cross-sectional data, we are hence able to disentangle unemployment-related changes in HCC from pre-existing differences in individual HCC levels. Second, we consider the context of unemployment by taking the unemployment duration (short- vs. medium-term) and the re-employment expectations during unemployment into account. In the process, we follow up on previous findings suggesting that differences in the uncertainty about one’s (future) employment situation explain why unemployed individuals feel more stressed than employed individuals^25^. Third, past studies found that HCC and perceived stress were generally not correlated^5^, indicating that the two stress measures capture different aspects of stress. In particular, it was suggested that cortisol secretion is closely linked to anticipation, stressor novelty, and social-evaluative threat^39–41^, whereas stress perceptions are more closely linked to stressor demands and cognitive appraisals^1^. We extend this line of research by contrasting changes in HCC and perceived stress in a large longitudinal study with unemployment as a potentially important real-world stressor.

## Results

HCC (in pg per mg hair) in the scalp-near 3 cm hair was determined as a measure of the average cortisol secretion within the last three months. As HCC data are typically not normally distributed and the conducted statistical analysis are sensitive to outliers, we winsorized and log-transformed the raw HCC data separately for each HCC collection wave (analogous to^41^). In a first step, we examined the stability of HCC over the five quarterly hair collection waves (see Table 1). The mean HCC levels decreased from the first HCC measurement occasion (Q1) to the second HCC measurement occasion (Q2) and remained fairly constant in later collection waves (Q3–Q5). The re-test correlations of HCC ranged from 0.37 to 0.66 underlining that HCC is highly stable over time. Still, there was substantial variation in the HCC levels as well as HCC changes. Nearly identical results were found based on individuals who provided valid hair samples during all five hair collection waves (see Table S1 in the supplementary materials).

**Table 1:** Descriptive statistics for HCC across the five collection points (Q1–Q5).

Measurement occasion	*N*_Obs_	Mean (HCC_*t*_)	Var (HCC_*t*_)	Var (HCC_*t*_–HCC_1_)	Re-test correlations			
					HCC_1_	HCC_2_	HCC_3_	HCC_4_
Q1	687	1.61	0.46					
Q2	424	1.36	0.48	0.43	0.53			
Q3	314	1.4	0.55	0.44	0.54	0.66		
Q4	268	1.43	0.39	0.43	0.47	0.37	0.51	
Q5	219	1.42	0.44	0.41	0.53	0.58	0.54	0.47

HCC: hair cortisol concentration (in pg per mg hair); *N*_Obs_: number of valid hair samples per measurement occasion; Mean(HCC_*t*_): mean of winsorized and log-transformed HCC values across measurement occasions; Var(HCC_*t*_): variance of winsorized and log-transformed HCC values across measurement occasions; Var(HCC_*t*_–HCC_1_): variance of winsorized and log-transformed HCC changes between the first measurement occasion (Q1) and measurement occasion *t.*

In order to investigate whether experiencing unemployment accounts for inter-individual differences in the intraindividual changes in HCC, we applied (latent) baseline change score modelling^42–44^. Figure S2 in the supplementary materials depicts a path diagram of the model. Besides the HCC data, we also integrated assessments of perceived stress obtained via monthly questionnaires into the model to also explore the effects of unemployment on perceived stress. To obtain a measure of perceived stress that corresponds to the same timeframe as the HCC data, we aggregated the scores of the stress items across the previous three months for measurement occasions Q2–Q5. For Q1 we used the responses to the stress items during the first measurement occasion. We investigated whether changes in HCC and perceived stress were related to the various employment transitions individuals could experience between Q1 (i.e., when all individuals were still employed) and later waves (i.e., when some individuals have entered unemployment).

Because the sample consists of workers who registered as jobseekers roughly three months before the first measurement occasion, most entries into unemployment occurred between Q1 and Q2. Moreover, due to the favorable labor market conditions at the time in which we conducted our study, most individuals who entered unemployment found a new job rather quickly so that only few individuals were still unemployed at Q4. In order to have sufficiently large sample sizes of unemployed individuals and to be able to examine the effects of unemployment duration, we thus limited the analyses to the first three hair collection waves (Q1–Q3) and excluded individuals who entered unemployment after Q2 (*N* = 19). This way, changes in HCC and perceived stress occurring between Q1 and Q2 can be linked to short-term unemployment (i.e., 1–3 months) and changes occurring between Q1 and Q3 can be linked to medium-term unemployment (i.e., 4–6 months). We further excluded individuals with missing values on the employment status (*N* = 148) and individuals who entered unemployment more than once (*N* = 19) between Q1 and Q3 (see Fig. S1 in the supplementary materials for participant flow chart).

**Figure 1:**
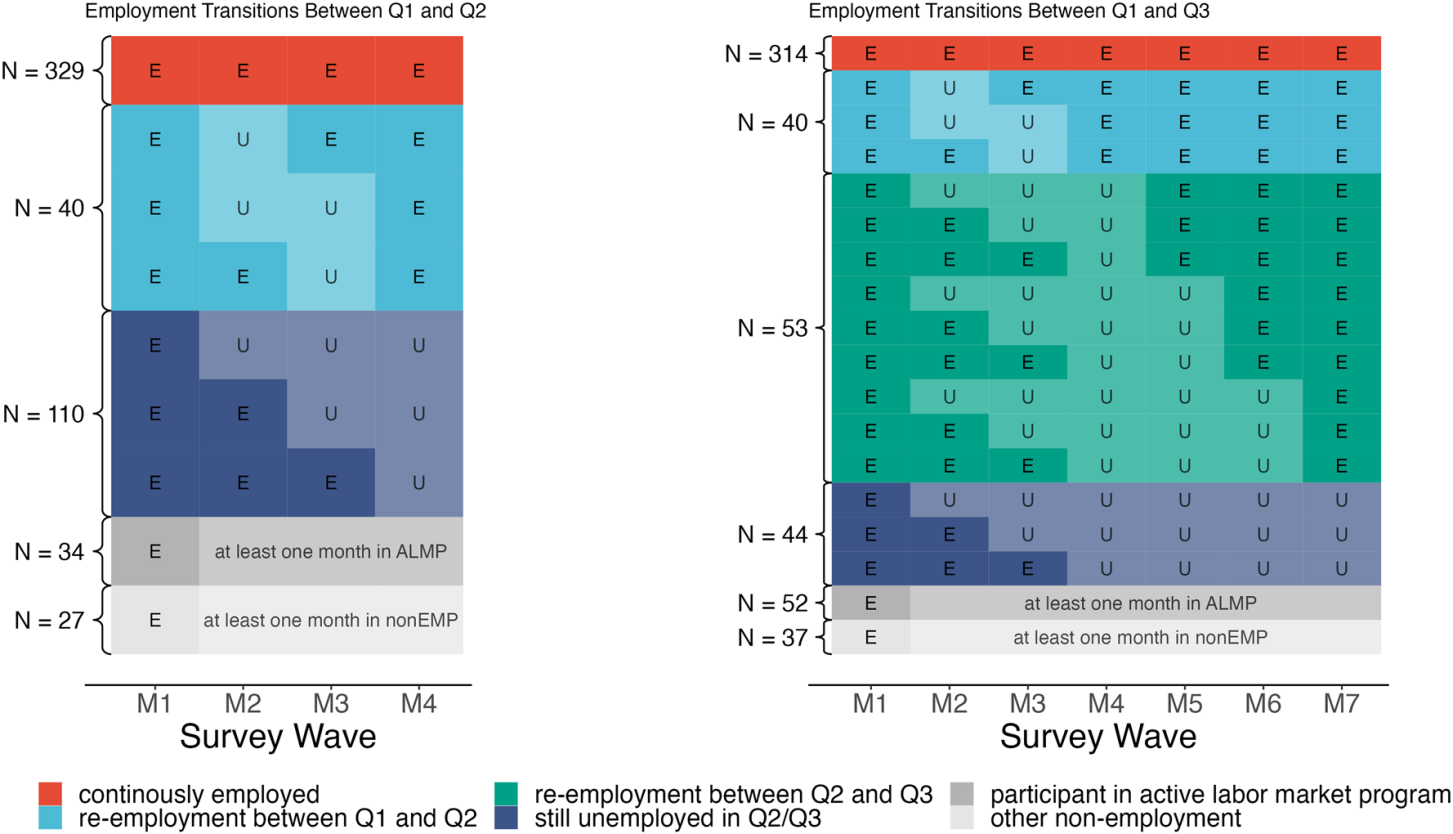
Employment patterns for the different employment groups (without considering re-employment expectations). E: Employed. U: Unemployed. M1–M7: monthly survey waves. Q1–Q3: quarterly hair collection waves. The sample sizes of the employment groups are presented next to the braces.

In our first main analysis, we examined general effects of becoming unemployed. Specifically, we grouped individuals according to their employment transitions in the period between Q1 and Q2 as well as Q1 and Q3. The resulting groups with their corresponding sample sizes are depicted in Fig. 1. By regressing the changes in HCC and perceived stress onto dummy variables corresponding to the various groups, we obtained the average group differences in respect to these changes. When deriving these average group differences, we controlled for all stable between-person differences affecting HCC and perceived stress by regressing the changes in HCC and perceived stress onto the initial mean-centered levels of these variables. Moreover, we regressed the initial levels of HCC and perceived stress (i.e., at Q1) onto these dummy variables to examine whether the groups already differed at the first measurement occasion. In all regressions, we statistically controlled for the self-reported reason for the registration as job seeker (mass layoff or plant closure vs. other reasons), age (grand-mean centered), gender, recruitment time (cohort 1 vs. cohort 2) as well as the season during which the hair sample was collected. We did not include the frequency of hair washing, hair dyeing and self-rated health in the model because these characteristics were not correlated with HCC.

**Figure 2:**
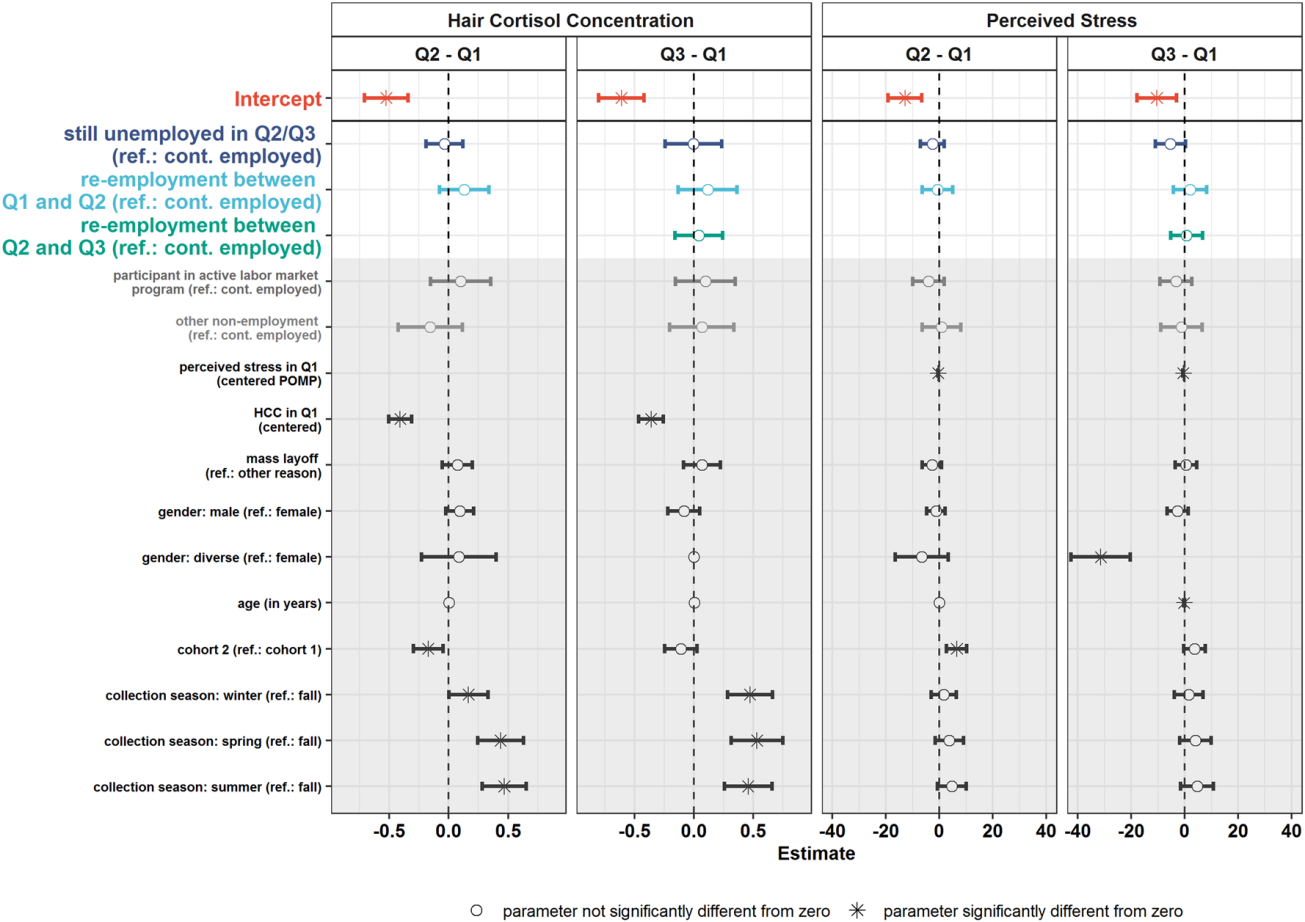
General effect of unemployment status on HCC and perceived stress. Q1–Q3: quarterly hair collection waves. The figure depicts the regression coefficients for the change variables in HCC (first two columns) and perceived stress (third and fourth column) between Q1 and Q2 (first and third column) and Q1 and Q3 (second and fourth column) with the corresponding 95%-confidence intervals. Hair cortisol levels (in pg per mg hair) were winsorized and log-transformed before analysis. The perceived stress values ranged from 0–100. Colors reference the four employment groups described in Fig. 1. The numerical values underlying this figure are presented in Table S2 in the supplementary materials. Full model results are available at https://osf.io/ex8ph/. A path diagram of the underlying model is presented in Fig. S2 in the supplementary materials.

After excluding individuals with missing values on the control variables (*N* = 14), the analyses were based on a sample of *N* = 526. The model fit the data well (χ^2^ = 108.682; *df* = 99; *p* = 0.238, RMSEA = 0.014 [0;0.028]; CFI = 0.998). The estimated regression coefficients for the changes in HCC and perceived stress are presented in Fig. 2. The full model results are available at: https://osf.io/ex8ph/. The average HCC levels of continuously employed individuals with zeros on all covariates decreased from Q1 to Q2 and from Q1 to Q3 (*Intercept*). Similarly, the average perceived stress rating (scale: 0–100) of continuously employed individuals decreased from Q1 to Q2 and from Q1 to Q3. The average changes in HCC and perceived stress occurring between Q1 and Q2 as well as between Q1 to Q3 of individuals who became unemployed between Q1 and Q2 were not statistically different from those of continuously employed individuals. This was the fact regardless of the examined length of unemployment (i.e., short- vs. medium-term unemployment) and the re-entry into paid employment. Moreover, the four employment groups did not significantly differ in their initial HCC or perceived stress levels when accounting for the various control variables (see Table S4 in the supplementary materials). The regression weights of the control variables are presented in Fig. 2 and Table S4.

**Figure 3:**
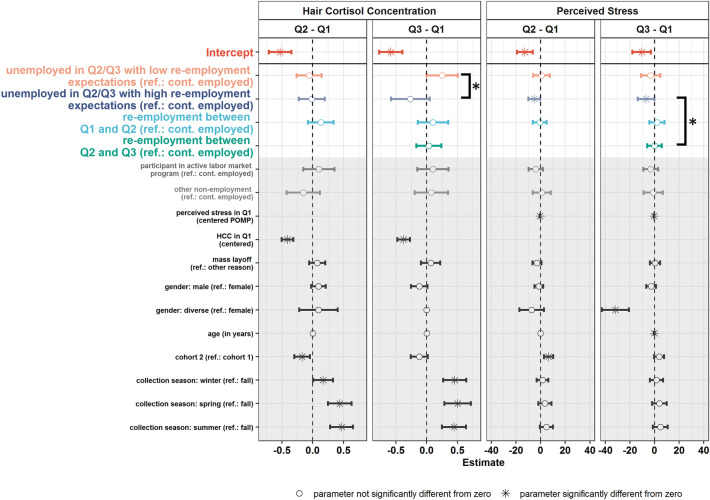
Effects of unemployment on HCC and perceived stress taking re-employment expectations into account. Q1–Q3: quarterly hair collection waves. The figure depicts the regression coefficients for the change variables in HCC (first two columns) and perceived stress (third and fourth column) between Q1 and Q2 (first and third column) and Q1 and Q3 (second and fourth column) with the corresponding 95%-confidence intervals. Hair cortisol levels (in pg per mg hair) were winsorized and log-transformed before analysis. The perceived stress values ranged from 0–100. Colors reference the five employment groups described in Fig. S3. Stars beside a square bracket indicate that the regression coefficients of two employment groups are significantly different from each other. The numerical values underlying this figure are presented in Fig. S2 in the supplementary materials. Full model results are available at https://osf.io/ex8ph/. A path diagram of the underlying model is presented in Fig. S2 in the supplementary materials.

In our second main analysis, we extended the previously described model by additionally considering the re-employment expectations during unemployment. In particular, we distinguished between unemployed individuals with high and with low re-employment expectations at Q2 and Q3 (see Fig. S3 in the supplementary materials for patterns and group sizes). This analysis relied on the same individuals as the previously reported model (*N* = 526) and the model fit was good (χ^2^ = 148.863; *df* = 111; *p* = 0.010, RMSEA = 0.025 [0.012;0.036]; CFI = 0.993). The main results are depicted in Fig. 3. Being short-term unemployed (i.e., changes between Q1 and Q2) was not associated with differential mean changes in HCC in comparison to continuously employed individuals regardless of the re-employment expectations. However, the average changes in HCC significantly differed between individuals who were unemployed in Q3 (i.e., who had been unemployed for 4–6 months) and reported *low* versus *high* re-employment expectations (*b* = − 0.517, *p* = 0.014). Moreover, individuals who were unemployed in Q3 and reported low re-employment expectations showed stronger mean increases in HCC between Q1 and Q3 compared to continuously employed individuals (*b* = 0.252, *p* = 0.05). However, this difference is not significantly different from zero with a *p*-value just above 0.05. Individuals who were still unemployed in Q3 and reported high re-employment expectations showed descriptively, albeit not statistically significant (*b* = − 0.265, *p* = 0.10), stronger mean decreases in HCC compared to continuously employed individuals. Moreover, the five employment groups did not significantly differ in their initial HCC levels when accounting for the various control variables (see Table S5 in the supplementary materials). The regression weights of the control variables are presented in Fig. 3 and Table S5.

In terms of perceived stress, individuals who were unemployed in Q2 with high re-employment expectations showed stronger mean decreases in perceived stress between Q1 and Q2 compared to continuously employed individuals (*b* = − 5.049, *p* = 0.04). The same was true for changes between Q1 and Q3 with unemployed individuals with high re-employment expectations showing stronger mean-level decreases in perceived stress compared to continuously employed individuals (*b* = − 6.86, *p* = 0.048). Further, the average changes in perceived stress significantly differed between individuals who were unemployed in Q3 with *high* re-employment expectations and individuals who became re-employed between Q2 and Q3 (*b* = − 8.811, *p* = 0.047). Moreover, individuals who became unemployed between Q1 and Q2 and were still unemployed in Q3 while reporting good re-employment prospects had significantly lower initial perceived stress levels (i.e., in Q1) compared to continuously employed individuals (*b* = − 10.829, *p* = 0.038; see Table S4 in the supplementary materials). Interestingly, (residual) correlations between initial levels as well as changes in HCC and perceived stress were not significantly different from zero in all models.

## Discussion

The present longitudinal study examined (i) whether short- and medium-term unemployment is associated with changes in hair cortisol concentration (HCC) as a biomarker of chronic stress and (ii) how re-employment expectations during unemployment moderate this association. Descriptive analyses revealed that even though rank-order stability of the HCC levels was high across the five quarterly hair collection waves, individuals meaningfully differed in their initial HCC levels as well as in their intraindividual changes over time. In line with previous research, this underlines that HCC levels are jointly influenced by stable dispositions as well as the changeable circumstances of life^45^.

The first key conclusion of our study is that an uncertain future of looming unemployment is even more stressful than unemployment itself. This implication originates from the observed mean-level decrease in HCC and perceived stress from the first measurement wave, when all individuals were insecurely employed, to later measurement waves, when some individuals entered unemployment whereas others remained employed. Thus, the months immediately *before* a potential loss of work, during which individuals experience high levels of uncertainty about their future and bear the high burden of fulfilling their work duties while looking for a new job, seem to be related to increased cortisol secretion and elevated levels of perceived stress. Once individuals gained more certainty about their employment situation, regardless of whether or not individuals actually entered unemployment, mean levels of HCC and perceived stress decreased again. Thus, stress decreased with resolving uncertainty, irrespective of whether it is resolved in a favorable or unfavorable manner. Highlight the role of (economic) uncertainty for health and are line with previous research showing that job insecurity can be a potent chronic stressor that has stronger detrimental effects in terms of stress and well-being than the actual job loss^46,47^. This can be explained by the fact that the uncertainty associated with job insecurity often inhibits the effective use of coping strategies^1,48^. For example, insecurely employed individuals will likely be more hesitant to look for a new job compared to individuals who already lost their jobs or who know for certain that they will lose their job in the future.

The second key conclusion of our study is that being out of work is not necessarily associated with higher HCC levels than being in work. Specifically, the results indicate that the current unemployment status is not related to changes in the cortisol system per se but that it is important to consider the context of unemployment, particularly future re-employment prospects. Whereas we found no general associations between the current unemployment status and changes in HCC, differentiating between unemployed individuals with high vs. low re-employment expectations yielded a more heterogeneous picture. In particular, medium-term unemployment (i.e., 4–6 months) was associated with a *stronger mean level increase* in HCC when re-employment expectations were low compared to when they were high. For short-term unemployment (i.e., 1–3 months) no such effects were present, regardless of the re-employment expectations. These results suggest that it is not so much the actual status of being unemployed but rather poor re-employment prospects during unemployment that are associated with elevated cortisol levels when unemployment persists. In consequence, unemployed individuals with poor re-employment prospects are an at-risk group of numerous negative health outcomes associated with increased cortisol secretion, notably cardiovascular disease^7^, hypertension^49^, or mental disorders^11–13^. Thus, our study has important practical implications for case-workers and policymakers as it emphasizes the need for targeted interventions that aim at promoting the health of individuals with low employability (e.g., by reintegrating them into the workforce).

Further, the lack of effects of short-term unemployment on HCC as well as the descriptively positive effects of unemployment on HCC, when re-employment prospects are good, provide additional evidence that being unemployed can also have several benefits compared to being employed (e.g., more leisure time, less work-related stress). That relates to a sizable body of studies showing that work can also be a major stressor^50–54^. Moreover, these findings are in line with research indicating that unemployment is associated with positive effects in terms of affective well-being (i.e., feeling happy) when re-employment expectations are high^55^. Interestingly, the literature on affective well-being also cites the burdens that come with working as a key reason for why most unemployed individuals are not clearly worse off compared to workers^27,56^. We, therefore, note that the associations between unemployment and both cortisol secretion as well as affective well-being are context-dependent in similar ways. In contrast, the average effects of unemployment on how individuals evaluate their lives (i.e., life satisfaction) are negative even if re-employment prospects are good^55,57^. Taken together, these findings underline that the effects of unemployment on cognitive well-being facets (e.g., life satisfaction) are more pronounced and generally negative but that effects of unemployment on daily lives seem to be highly context-dependent and can even be positive, leading to less physiological stress and higher affective well-being.

We also examined whether short- and medium-term unemployment was related to changes in perceived stress. Analogously to HCC, we found no evidence of general effects of the current unemployment status on perceived stress. Crucially, however, when taking future re-employment expectations into account, unemployment was differentially related to perceived stress and HCC. In particular, mean self-reported stress levels of unemployed individuals who reported high re-employment expectations decreased more strongly than those of continuously employed individuals. This result is, again, in line with previous findings suggesting that the increased uncertainty of one’s employment situation matters most for perceived stress^25^. A striking difference between the results for HCC and perceived stress is that no average differences in perceived stress were found for individuals in medium-term unemployment (i.e., 4–6 months) who report high as opposed to low re-employment expectations. Thus, we conclude that HCC and perceived stress are differentially related to unemployment, when taking re-employment expectations into account. Further, in line with numerous existing studies^5^, the initial levels of HCC and perceived stress as well as the subsequent changes in HCC and perceived stress were not correlated. Taken together, this study therefore underlines that HCC and perceived stress capture different aspects of stress. Nevertheless, more multimethod studies incorporating both cortisol data and self-reported stress are needed to better understand why (long-term) cortisol secretion and perceived stress are differentially affected by unemployment. This would be important to better understand the channels by which long-term health outcomes are affected by unemployment.

## Limitations and future directions

The longitudinal data of the present study made it possible to disentangle unemployment-related changes in HCC and perceived stress while controlling for pre-existing differences in these stress measures. However, there might be time-variant variables that drive the observed effects so that the identified effects do not necessarily represent causal effects. To effectively probe the causal nature of the effects, studies exploiting entirely exogenous uncertainty and unemployment shocks would be needed. Moreover, most individuals who entered unemployment in the present study only stayed unemployed for a few months. Thus, we did not examine the effects of long-term unemployment. To this end, a longitudinal study that targets individuals who are at a high risk for long-term unemployment would be needed. Such a long-running study would further be highly valuable to examine the role of HCC and perceived stress for predicting the negative health effects that have been associated with unemployment in previous studies.

## Materials and methods

### Data

The utilized data comes from the German Job Search Panel (GJSP^58^), a monthly app-based panel study with five quarterly hair collection waves for measuring HCC. To recruit employed German jobseekers who are at high risk of losing their job, the GJSP exploits the German job search registration process, which requires employees to register as jobseekers at least three months prior to their expected job loss. Crucially, only around 60% of individuals who register as jobseekers enter unemployment later on, whereas the other individuals manage to keep their jobs or to immediately start a new job without entering unemployment^59^. GJSP participants were recruited as two cohorts. For the first cohort, 79,710 jobseekers who were likely to be affected by mass layoffs and 48,126 jobseekers who were likely to lose their jobs due to other reasons were identified between November 2017 and May 2019. For the second cohort, 42,340 jobseekers all of whom were likely to be affected by mass layoffs were identified between July 2020 and February 2021. Identified jobseekers were contacted via letter or email^60^. In total, 6591 individuals (N_cohort1_ = 4700, N_cohort2_ = 1891) started filling out the entry survey and 2449 individuals were included in the final sample (for exclusion criteria see^58^). Analyses based on data of the first cohort indicate that the overall selection bias of the sample was small despite the low sign-up rate^58^. Survey data was gathered via a specifically developed smartphone app, which ran on Android and iOS (for details see^61^). Over up to 25 months, participants received monthly questionnaires assessing a wide range of information, including perceived stress (for details see^58^). The parallel collection of hair samples ran on a quarterly basis from the beginning of study participation for up to 1 year. Previous analyses based on the first GJSP cohort showed that effects of selective participation in the hair collection were only small^58^.

The study protocol was approved on Dec 13, 2017, by the ethics committee of the Department of Education and Psychology at Freie Universität Berlin and informed consent was obtained from all respondents at the start of the entry survey. The survey was carried out in accordance with all relevant guidelines and regulations.

### Measures

The wordings of all utilized questionnaire items are presented in Materials S1 in the supplementary materials.

#### Hair cortisol

On the seventh measurement day of survey waves 1, 4, 7, 10, and 13, individuals were asked via the survey app whether they were willing to send in a hair sample for HCC analysis. Respondents who indicated that (a) their hair was shorter than 2 cm or (b) that they took cortisone-based medication were excluded from the hair collection as non-eligible. Individuals who were not willing or eligible for the cortisol study in the first hair collection wave were excluded from later hair collection waves. Eligible respondents received the hair collection kits via mail and were asked to send in three hair strands of 3 mm diameter each to the research team within 10 days after receiving the collection kit. When insufficient hair material was sent in (i.e., less than 3 cm hair or less than 7.5 g hair), HCC data was treated as missing.

For each hair sample, respondents received a 10 euros cash incentive. Moreover, individuals could receive feedback concerning their hair cortisol levels at the end of the study. The 3 cm hair segments closest to the scalp were analyzed by the bio laboratory Dresden Lab Service using immunoassays to obtain a measure of the cumulated cortisol exposure over the last three months. As a quality check, 10% of the hair samples were analyzed using liquid chromatography tandem-mass spectrometry (LC–MS/MS). The HCC values obtained from both analysis methods correlate between 0.95 and 0.999 across the five hair collection waves. Because no thresholds for healthy/unhealthy HCC levels have been established, they can primarily be compared between different groups within a study^62^.

#### Self-reported stress

Self-reported stress was assessed monthly via the survey app by asking respondents to indicate how often they felt ‘stressed’ and ‘overburdened’ within the last week. Individuals could respond to both items on a five-point rating scale ranging from (1) *rarely or none of the time (less than 1 day)*, (2) *some or a little of the time (1–2 days)*, (3) *occasionally or a moderate amount of time (3–4 days)*, (4) *most or all of the time (5–7 days)*, to (5) *don’t know*. *Don’t know* answers were coded as missing values. To obtain a measure of self-reported stress that corresponds to the same time frame as the HCC values, we averaged the scores of the stress item across the concurrent and two previous survey months. Moreover, we transformed the item scores into percent of maximum possible scores (POMP^63^) so that they range from 0 to 100 and can be interpreted in terms of percentage points.

#### Employment groups

Each survey month, respondents were asked about their current employment status. Individuals were categorized as being *employed* when they were employed or self-employed, and as *unemployed* when they were unemployed. Moreover, individuals who took part in public subsidy programs or occupational retraining were categorized as *participants of active labor market policies (ALMPs)*. Individuals who were in occupational training, school, or university, unable to work (i.e., due to illness), or retired were categorized as *individuals with other non-employment*. The same was true for individuals who chose the category ‘other’ when answering the question on their employment situation. In survey months, in which individuals were not employed, they were asked to respond to the question “How likely is it that you will start a paid job within the next three months?” using an 11-point rating scale ranging from 0 to 100%. We defined the high vs. low re-employment groups by dichotomizing this variable (0–50%: low re-employment expectation vs. 60–100%: high re-employment expectation).

### Control variables

Age and gender were assessed during the entry survey. Gender was assessed with the following three categories: *female*, *male*, and *other*. Age (in years) was mean-centered for the analyses. Moreover, we differentiated between individuals who reported that they registered as job seeking due to (a) mass layoffs and plant closures or (b) other reasons. Lastly, we controlled for the different recruitment times (cohort 1 vs. cohort 2) and the season during which the first hair sample was collected. For individuals who did not send in a valid hair sample in Q1 but sent in a hair sample in later collection waves, we imputed the time point at which individuals would have sent in their first hair samples (e.g., by subtracting three months from the date at which they send in their second hair sample).

### Statistical model

We applied (latent) baseline change score models^42–44^ to model the initial levels of HCC and perceived stress as well as the changes in these constructs between Q1 and Q2 as well as Q1 and Q3. We used the two (aggregated) items of perceived stress to define the true (i.e., error-free) stress levels and the true intra-individual changes using latent variables. Because change score modeling requires strong measurement invariance^64^, we constrained the factor loadings and the intercepts of the stress items to be invariant over time. We included an indicator-specific factor to account for indicator-specific variance of the second stress item over time^65,66^. To control for differences in the initial HCC and perceived stress levels, we regressed the HCC changes onto the HCC levels at Q1 and the changes in perceived stress onto the levels of perceived stress at Q1^42^. Moreover, we regressed the change variables as well as the variables corresponding to the initial levels of HCC and perceived stress onto dummy variables denoting the various employment groups as well as the control variables. A path diagram of the utilized model is depicted in Fig. S2 in the supplementary materials. All models were fitted using the structural equation modeling software lavaan (version 0.6-12;^67^) in R (version 4.2.1;^68^). We used the robust full information maximum likelihood estimator in order to handle missing data and to account for the nonnormal distribution of the indicators while utilizing all available information^69^.

## Supplementary Information

Supplementary Information.

## Data Availability

All scripts and model results are available at the online repository of this study (https://osf.io/ex8ph/). The data are available for research purposes upon request and can be obtained by contacting the corresponding author.
