# Supplementary material for: Unemployment and hair cortisol as a biomarker of chronic stress
Source: Sci Rep. 2022 Dec 14;12:21573. doi: 10.1038/s41598-022-25775-1 (PMC9751113; doi:10.1038/s41598-022-25775-1)
Supplement: Supplementary file 1 — Supplementary Information. [file 41598_2022_25775_MOESM1_ESM.pdf]

Unemployment and Hair Cortisol as a Biomarker of Chronic Stress

Lawes, Hetschko, Schöb, Stephan, and Eid

Material S1: Questionnaire Items

Self-Reported Stress

Last week… [„In der vergangen Woche…"]

1. I felt overburdened [„habe ich mich überlastet gefühlt."]
2. I felt stressed [„habe ich mich gestresst gefühlt."]

Scale: rarely or none of the time (less than 1 day) (1), some or a little of the time (1-2 days) (2), occasionally or a moderate amount of time (3-4 days) (3) and most or all of the time (5-7 days) (4).

Employment Status

Source: 'The Value of Work' (see Knabe et al., 2010)

Which of the following main occupations applies to you? (Please choose only one option!) ["*Welche der nachfolgenden Haupttätigkeiten trifft auf Sie zu?*"]

- Part-time or full-time work with at least 15 working hours per week ["Teilzeit- oder Vollzeitbeschäftigung mit mindestens 15 Stunden pro Woche"]
- Part-time work with less than 15 working hours per week ["Teilzeitbeschäftigung mit unter 15 Stunden pro Woche"]
- Self-employed with at least 15 working hours per week ["Selbständig mit mindestens 15 Stunden pro Woche"]
- Public employment measure (e.g., One-euro job) ["öffentliche Beschäftigungsmaßnahme (z.B. Ein-Euro-Job)"]
- Registered as unemployed (and not participating in a support scheme) ["Arbeitslos gemeldet (und nicht in einer Fördermaßnahme)"]
- Seeking employment and participating in an educational programme or other support scheme ["Arbeitssuchend und in einer Fortbildungs- oder sonstigen Fördermaßnahme"]
- Early retirement/ old-age pensioner ["Vorruhestand/Altersrentner"]
- In training/studying ["in Ausbildung/Studium"]
- Other form of non-employment ["sonstige Nicht-Erwerbstätigkeit"]

Re-Employment Expectations

Source: SOEP (for original items see TNS Infratest Sozialforschung, 2015):

How likely is it that the following changes to your professional life will occur within the next three months? [Wie wahrscheinlich ist es, dass innerhalb der nächsten drei Monate die folgenden beruflichen Veränderungen für Sie eintreten]

1. Start a paid job? ["Dass Sie eine bezahlte Beschäftigung aufnehmen?"]

Scale: 11-point-scale from 0%, 10%, 20%, ... to 100%

Table S1

Descriptive Statistics for HCC across the Five Collection Points for Complete Cases

Measurement Occasion	N_{Obs}	Mean(HCC_t)	Var(HCC_t)	Var(HCC_t - HCC_1)	Re-Test Correlations			
					HCC_1	HCC_2	HCC_3	HCC_4
Q1	131	1.62	0.45					
Q2	131	1.4	0.47	0.52	.43			
Q3	131	1.38	0.49	0.45	.53	.66		
Q4	131	1.37	0.34	0.41	.49	.33	.49	
Q5	131	1.48	0.44	0.45	.5	.57	.54	.49

Notes. HCC: hair cortisol concentration (in pg per mg hair). N_{Obs}: Number of valid hair samples per measurement occasion. Mean(HCC_t): Mean of winsorized and log-transformed HCC values across measurement occasions. Var(HCC_t): Variance of winsorized and log-transformed HCC values across measurement occasions. Var(HCC_t - HCC_1): Variance of winsorized and log-transformed HCC changes between the first measurement occasion (Q1) and measurement occasion *t*.

Table S2

General Effect of Unemployment Status on HCC and Perceived Stress

	Hair Cortisol Concentration		Perceived Stress	
	Q2 – Q1	Q3 – Q1	Q2 – Q1	Q3 – Q1
Intercept	**-0.526** [-0.71;-0.342] (*p* < .001)	**-0.611** [-0.803;-0.42] (*p* < .001)	**-12.904** [-19.272;-6.536] (*p* < .001)	**-10.446** [-17.922;-2.969] (*p* = .006)
re-employment between Q1 and Q2 (ref.: cont. employed)	0.131 [-0.076;0.339] (*p* = .215)	0.115 [-0.133;0.362] (*p* = .363)	-0.714 [-6.386;4.957] (*p* = .805)	1.964 [-4.255;8.183] (*p* = .536)
re-employment between Q2 and Q3 (ref.: cont. employed)	-	0.041 [-0.16;0.242] (*p* = .687)	-	0.7 [-5.288;6.689] (*p* = .819)
still unemployed in Q2/Q3 (ref.: cont. employed)	-0.035 [-0.189;0.119] (*p* = .654)	-0.004 [-0.243;0.234] (*p* = .971)	-2.645 [-7.089;1.8] (*p* = .243)	-5.39 [-11.029;0.248] (*p* = .061)
participant in active labor market program (ref.: cont. employed)	0.1 [-0.154;0.355] (*p* = .44)	0.095 [-0.157;0.348] (*p* = .458)	-4.04 [-9.898;1.818] (*p* = .176)	-3.297 [-9.213;2.619] (*p* = .275)
other non-employment (ref.: cont. employed)	-0.155 [-0.426;0.116] (*p* = .262)	0.066 [-0.205;0.336] (*p* = .633)	0.811 [-6.481;8.103] (*p* = .827)	-1.24 [-9.011;6.532] (*p* = .755)
perceived stress in Q1 (centered POMP)	-	-	**-0.501** [-0.572;-0.429] (*p* < .001)	**-0.552** [-0.634;-0.471] (*p* < .001)
HCC in Q1 (centered)	**-0.409** [-0.504;-0.314] (*p* < .001)	**-0.362** [-0.466;-0.258] (*p* < .001)	-	-
mass layoff (ref.: other reason)	0.073 [-0.054;0.201] (*p* = .261)	0.068 [-0.088;0.224] (*p* = .393)	-2.785 [-6.447;0.878] (*p* = .136)	0.445 [-3.624;4.514] (*p* = .83)
gender: male (ref.: female)	0.092 [-0.025;0.209] (*p* = .125)	-0.085 [-0.22;0.049] (*p* = .215)	-1.267 [-4.756;2.222] (*p* = .477)	-2.655 [-6.611;1.302] (*p* = .188)
gender: diverse (ref.: female)	0.086 [-0.228;0.4] (*p* = .591)	0 [0;0] (*p* = 1)	-6.552 [-16.503;3.399] (*p* = .197)	**-31.448** [-42.507;-20.388] (*p* < .001)
age (in years)	0.002 [-0.004;0.009] (*p* = .458)	0.002 [-0.005;0.009] (*p* = .596)	-0.004 [-0.162;0.154] (*p* = .96)	**-0.234** [-0.414;-0.054] (*p* = .011)
cohort 2 (ref.: cohort 1)	**-0.172** [-0.298;-0.047] (*p* = .007)	-0.111 [-0.248;0.027] (*p* = .115)	**6.51** [2.737;10.283] (*p* < .001)	3.723 [-0.353;7.8] (*p* = .073)
collection season: winter (ref.: fall)	**0.165** [0.001;0.33] (*p* = .049)	**0.471** [0.282;0.66] (*p* < .001)	1.564 [-3.146;6.274] (*p* = .515)	1.49 [-3.839;6.819] (*p* = .584)
collection season: spring (ref.: fall)	**0.437** [0.243;0.631] (*p* < .001)	**0.531** [0.312;0.749] (*p* < .001)	3.711 [-1.606;9.027] (*p* = .171)	4.021 [-1.902;9.943] (*p* = .183)
collection season: summer (ref.: fall)	**0.468** [0.281;0.654] (*p* < .001)	**0.456** [0.255;0.656] (*p* < .001)	4.668 [-0.692;10.028] (*p* = .088)	4.616 [-1.51;10.742] (*p* = .14)

Notes. Q1 – Q3: quarterly hair collection waves. 95%-confidence intervals are presented in brackets and the two-sided *p* values in parentheses. When coefficients are printed in bold, their 95%-confidence intervals do not contain zero.

Table S3

Effects of Unemployment on HCC and Perceived Stress Taking Re-Employment Expectations into Account

	Hair Cortisol Concentration		Perceived Stress	
	Q2 – Q1	Q3 – Q1	Q2 – Q1	Q3 – Q1
Intercept	**-0.529** [-0.713;-0.345] (*p* < .001)	**-0.582** [-0.772;-0.392] (*p* < .001)	**-12.799** [-19.183;-6.415] (*p* < .001)	**-10.457** [-17.922;-2.991] (*p* = .006)
unemployed in Q2/Q3 with low re-employment expectations (ref.: cont. employed)	-0.058 [-0.261;0.145] (*p* = .573)	0.252 [0;0.504] (*p* = .05)	0.777 [-5.965;7.52] (*p* = .821)	-3.125 [-10.889;4.639] (*p* = .43)
unemployed in Q2/Q3 with high re-employment expectations (ref.: cont. employed)	-0.017 [-0.229;0.195] (*p* = .875)	-0.265 [-0.58;0.051] (*p* = .1)	**-5.049** [-9.865;-0.234] (*p* = .04)	**-6.86** [-13.668;-0.052] (*p* = .048)
re-employment between Q1 and Q2 (ref.: cont. employed)	0.131 [-0.076;0.339] (*p* = .215)	0.103 [-0.145;0.351] (*p* = .414)	-0.722 [-6.404;4.96] (*p* = .803)	1.95 [-4.277;8.177] (*p* = .539)
re-employment between Q2 and Q3 (ref.: cont. employed)	-	0.036 [-0.169;0.24] (*p* = .732)	-	0.006 [-5.862;5.874] (*p* = .998)
participant in active labor market program (ref.: cont. employed)	0.1 [-0.153;0.354] (*p* = .439)	0.101 [-0.151;0.353] (*p* = .431)	-3.913 [-9.753;1.928] (*p* = .189)	-3.092 [-9.045;2.861] (*p* = .309)
other non-employment (ref.: cont. employed)	-0.154 [-0.426;0.118] (*p* = .267)	0.076 [-0.196;0.348] (*p* = .585)	0.937 [-6.338;8.212] (*p* = .801)	-1.12 [-8.856;6.616] (*p* = .777)
perceived stress in Q1 (centered POMP)	-	-	**-0.499** [-0.57;-0.428] (*p* < .001)	**-0.554** [-0.635;-0.472] (*p* < .001)
HCC in Q1 (centered)	**-0.41** [-0.506;-0.315] (*p* < .001)	**-0.374** [-0.477;-0.272] (*p* < .001)	-	-
mass layoff (ref.: other reason)	0.074 [-0.055;0.203] (*p* = .261)	0.065 [-0.091;0.221] (*p* = .413)	-2.92 [-6.567;0.728] (*p* = .117)	0.356 [-3.718;4.43] (*p* = .864)
gender: male (ref.: female)	0.093 [-0.027;0.212] (*p* = .128)	-0.118 [-0.253;0.016] (*p* = .084)	-1.438 [-4.929;2.052] (*p* = .419)	-2.734 [-6.68;1.213] (*p* = .175)
gender: diverse (ref.: female)	0.092 [-0.22;0.404] (*p* = .562)	0 [0;0] (*p* = 1)	-7.273 [-17.263;2.717] (*p* = .154)	**-31.775** [-42.799;-20.75] (*p* < .001)
age (in years)	0.003 [-0.004;0.009] (*p* = .407)	0.001 [-0.006;0.008] (*p* = .832)	-0.025 [-0.186;0.135] (*p* = .758)	**-0.24** [-0.419;-0.061] (*p* = .008)
cohort 2 (ref.: cohort 1)	**-0.172** [-0.298;-0.047] (*p* = .007)	-0.12 [-0.256;0.017] (*p* = .086)	**6.487** [2.744;10.23] (*p* < .001)	3.702 [-0.359;7.763] (*p* = .074)
collection season: winter (ref.: fall)	**0.169** [0.005;0.332] (*p* = .043)	**0.454** [0.267;0.642] (*p* < .001)	1.562 [-3.131;6.255] (*p* = .514)	1.491 [-3.832;6.813] (*p* = .583)
collection season: spring (ref.: fall)	**0.439** [0.247;0.631] (*p* < .001)	**0.501** [0.286;0.717] (*p* < .001)	3.525 [-1.784;8.834] (*p* = .193)	3.98 [-1.924;9.884] (*p* = .186)
collection season: summer (ref.: fall)	**0.47** [0.284;0.656] (*p* < .001)	**0.445** [0.249;0.641] (*p* < .001)	4.66 [-0.681;10.001] (*p* = .087)	4.635 [-1.477;10.747] (*p* = .137)

Notes. Q1 – Q3: quarterly hair collection waves. 95%-confidence intervals are presented in brackets and the two-sided *p* values in parentheses. When coefficients are printed in bold, their 95%-confidence intervals do not contain zero.

Table S4

Differences at the First Measurement Occasion (Q1) for Employment Groups Without Taking the Re-Employment Expectations into Account

	Hair Cortisol Concentration	Perceived Stress
Intercept	**0.273** [0.117;0.43] (p < .001)	0[a] [0;0] NA
re-employment between Q1 and Q2 (ref.: cont. employed)	0.016 [-0.224;0.256] (p = .896)	0.257 [-9.864;10.378] (p = .96)
re-employment between Q2 and Q3 (ref.: cont. employed)	-0.075 [-0.265;0.114] (p = .437)	0.053 [-8.064;8.171] (p = .99)
still unemployed in Q3 (ref.: cont. employed)	-0.159 [-0.371;0.054] (p = .143)	1.964 [-8.362;12.29] (p = .709)
participant in active labor market program (ref.: cont. employed)	0.023 [-0.17;0.216] (p = .812)	-0.301 [-9.444;8.843] (p = .949)
other non-employment (ref.: cont. employed)	0.105 [-0.142;0.352] (p = .404)	7.608 [-4.027;19.243] (p = .2)
mass layoff (ref.: other reason)	-0.005 [-0.132;0.121] (p = .932)	**-6.635** [-12.351;-0.918] (p = .023)
gender: male (ref.: female)	0.084 [-0.035;0.202] (p = .167)	**-10.133** [-15.457;-4.809] (p < .001)
gender: diverse (ref.: female)	**0.585** [0.315;0.855] (p < .001)	**31.807** [18.388;45.226] (p < .001)
age (in years)	0.005 [-0.002;0.011] (p = .139)	-0.248 [-0.515;0.019] (p = .069)
cohort 2 (ref.: cohort 1)	0.043 [-0.079;0.165] (p = .488)	-4.491 [-10.215;1.234] (p = .124)
collection season: winter (ref.: fall)	**-0.333** [-0.489;-0.177] (p < .001)	-1.992 [-9.51;5.526] (p = .604)
collection season: spring (ref.: fall)	**-0.552** [-0.721;-0.383] (p < .001)	-4.788 [-13.043;3.466] (p = .256)
collection season: summer (ref.: fall)	**-0.263** [-0.443;-0.084] (p = .004)	-5.71 [-14.223;2.802] (p = .189)

Notes. [a] The intercept of the latent perceived stress variable was not estimated but set to zero in order to identify the model. 95%-confidence intervals are presented in brackets and the two-sided p values in parentheses. When coefficients are printed in bold, their 95%-confidence intervals do not contain zero.

Table S5

Differences at the First Measurement Occasion (Q1) for Employment Groups Taking the Re-Employment Expectations into Account

	Hair Cortisol Concentration	Perceived Stress
Intercept	**0.274** [0.117;0.43] ($p < .001$)	0 [a] [0;0] NA
unemployed in Q3 with low re-employment expectations (ref.: cont. employed)	-0.138 [-0.403;0.128] ($p = .309$)	13.098 [-2.333;28.529] ($p = .096$)
unemployed in Q3 with high re-employment expectations (ref.: cont. employed)	-0.182 [-0.499;0.135] ($p = .259$)	**-10.829** [-21.041;-0.618] ($p = .038$)
re-employment between Q1 and Q2 (ref.: cont. employed)	0.016 [-0.224;0.256] ($p = .897$)	0.256 [-9.903;10.414] ($p = .961$)
re-employment between Q2 and Q3 (ref.: cont. employed)	-0.075 [-0.265;0.115] ($p = .438$)	0.308 [-7.804;8.42] ($p = .941$)
participant in active labor market program (ref.: cont. employed)	0.024 [-0.169;0.217] ($p = .81$)	-0.12 [-9.29;9.049] ($p = .979$)
other non-employment (ref.: cont. employed)	0.105 [-0.142;0.352] ($p = .404$)	7.687 [-3.914;19.288] ($p = .194$)
mass layoff (ref.: other reason)	-0.006 [-0.132;0.121] ($p = .926$)	**-6.928** [-12.619;-1.237] ($p = .017$)
gender: male (ref.: female)	0.083 [-0.035;0.201] ($p = .17$)	**-10.476** [-15.751;-5.201] ($p < .001$)
gender: diverse (ref.: female)	**0.584** [0.315;0.854] ($p < .001$)	**31.222** [17.787;44.656] ($p < .001$)
age (in years)	0.005 [-0.002;0.011] ($p = .141$)	-0.267 [-0.533;0] ($p = .05$)
cohort 2 (ref.: cohort 1)	0.043 [-0.079;0.165] ($p = .491$)	-4.72 [-10.393;0.953] ($p = .103$)
collection season: winter (ref.: fall)	**-0.333** [-0.489;-0.177] ($p < .001$)	-1.853 [-9.282;5.576] ($p = .625$)
collection season: spring (ref.: fall)	**-0.552** [-0.721;-0.384] ($p < .001$)	-4.836 [-12.95;3.278] ($p = .243$)
collection season: summer (ref.: fall)	**-0.262** [-0.442;-0.082] ($p = .004$)	-5.235 [-13.701;3.231] ($p = .226$)

Notes. [a] The intercept of the latent perceived stress variable was not estimated but set to zero in order to identify the model. 95%-confidence intervals are presented in brackets and the two-sided p values in parentheses. When coefficients are printed in bold, their 95%-confidence intervals do not contain zero.

Figure S1

Participant Flow Chart

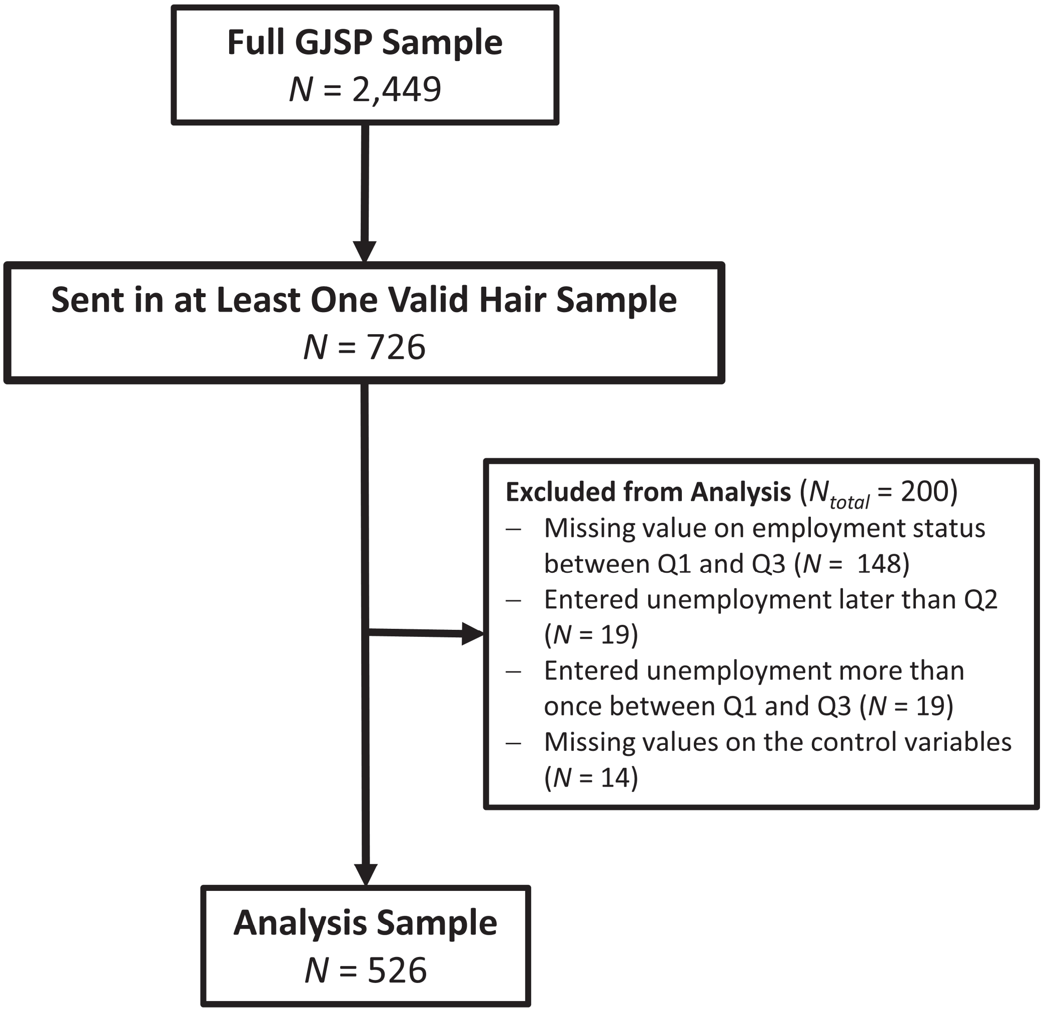

Note. GJSP: German Job Search Panel. Q1: first hair collection wave. Q3: third hair collection wave.

Figure S2

Analysis Model

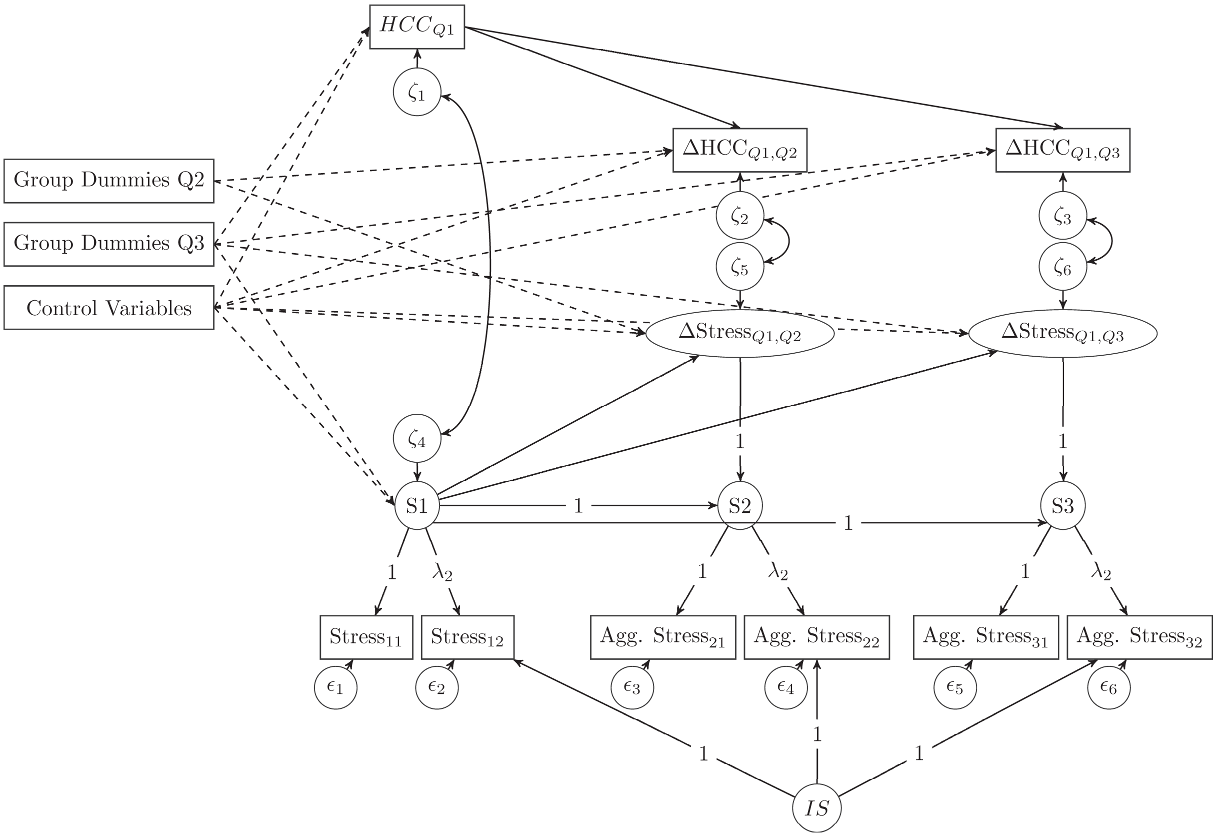

Note. The figure depicts the path model of the baseline change analysis. $Stress_{11}$ and $Stress_{12}$ represent the stress items measured at Q1. *Agg.* $Stress_{21}$ and *Agg.* $Stress_{22}$ represent the aggregated stress items measured at the survey measurement occasions 2, 3 and 4. *Agg.* $Stress_{31}$ and *Agg.* $Stress_{32}$ represent these items measured at the survey measurement occasions 5, 6 and 7. ε_1- ε_6 denote the measurement error of the (aggregated) stress items. *IS* is an indicator-specific factor for the second stress item. Factors *S1-S3* are the stress levels at Q1-Q3. The factors $\Delta Stress_{Q1,Q2}$ and $\Delta Stress_{Q1,Q3}$ capture changes in stress levels between Q1 and Q2 as well as Q1 and Q3, respectivly. HCC_{Q1} references the winsorized and log-transformed hair cortisol levels at Q1. $\Delta HCC_{Q1,Q2}$ and $\Delta HCC_{Q1,Q3}$ represent changes in HCC levels between Q1 and Q2 as well as Q1 and Q3, respectivly. Group dummies indicate the different employment groups (see Figure S3 and S4). The included control variables are described in the text. $\zeta_1 – \zeta_6$ depict residual variables of the stress and HCC levels as well as changes not predicted by the baseline measures, control variables or group dummies. The regression coefficients and variances are not depicted. Full model results can be found at https://osf.io/ex8ph/.

Figure S3

Employment Patterns for the Different Employment Groups (with re-employment expectations)

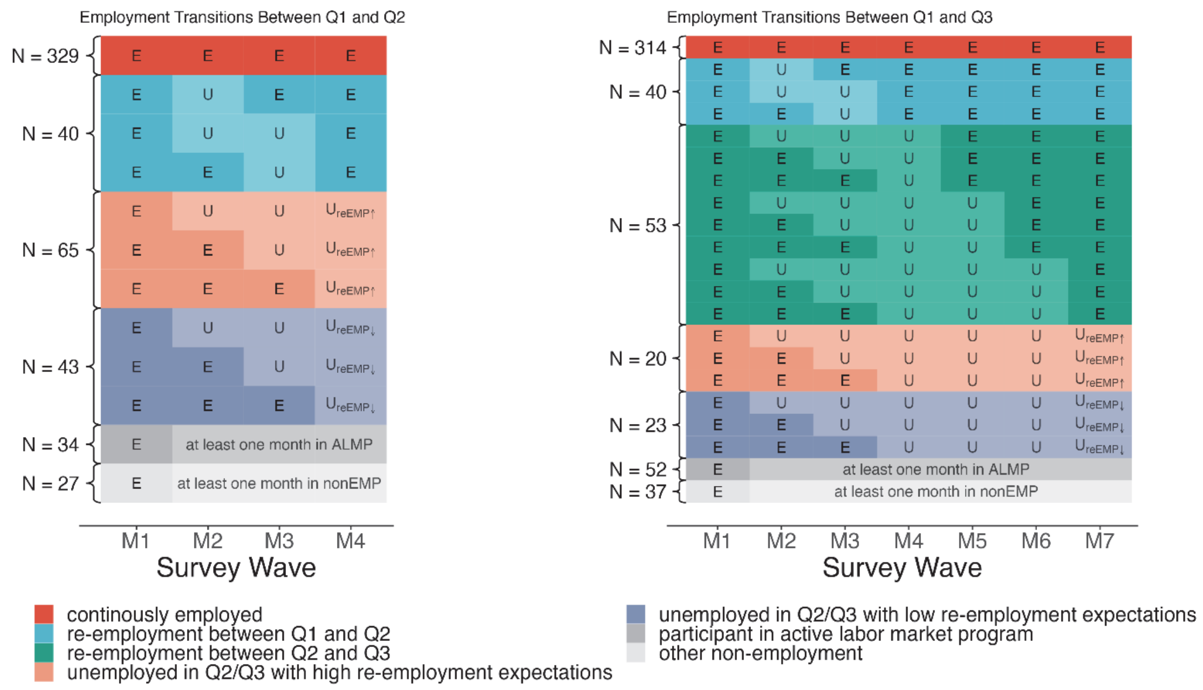

Note. E: Employed. U: Unemployed. M1 – M7: Monthly survey waves. Q1 – Q3: Quarterly hair collection waves. The sample sizes of the employment groups are presented next to the braces.

References

Knabe, A., Rätzel, S., Schöb, R., & Weimann, J. (2010). Dissatisfied with life but having a good day: Time-use and well-being of the unemployed. *Economic Journal*, *120*(547), 867–889. https://doi.org/10.1111/j.1468-0297.2009.02347.x

TNS Infratest Sozialforschung. (2015). SOEP 2015 – Erhebungsinstrumente 2015 (Welle 32) des Sozio-oekonomischen Panels: Personenfragebogen, Altstichproben. In *SOEP Survey Papers* (Nr. 274; Series A).
